# From Thomas Mann to John Green, how authors and books shape our understanding of TB

**DOI:** 10.5588/ijtldopen.25.0806

**Published:** 2026-03-13

**Authors:** M. Pai

**Affiliations:** 1 McGill School of Population and Global Health, McGill University, Montreal, QC, Canada;; 2 McGill International Tuberculosis Centre, Montreal, QC, Canada.

**Keywords:** tuberculosis, lung health, books, authors, stories, movies

## Abstract

Over the centuries, from Thomas Mann to John Green, many authors have attempted to tell the story of TB, or stories of people with TB, via books, poems, art, and films. Books, in particular, have helped us better understand the disease, its history, and its impact on people. This Editorial is a celebration of a selection of books and authors who have made important contributions, broadly grouped under four categories: historical; fiction; personal narratives; contemporary. Collectively, these books underscore the fact that TB has always thrived on racism, prejudice, poverty, and injustice, and continues to do so, even today. The book about the end of TB, sadly, is yet to be written.

TB is a plague that simply refuses to go away. Centuries before Robert Koch identified the causative bacterium in 1882, the disease killed people during the Neolithic age (about 10,000 years ago). Even today, it kills over a million people every year,^1^ and our ability to end this epidemic has taken a huge hit with the 2025 funding cuts by the United States and other western nations.^2^ The struggle continues.

From Thomas Mann to John Green, many authors have attempted to tell the story of TB, or stories of people with TB, via books, poems, art, and films. John Keats died of TB at the age of 25 and also lost his brother and mother to TB. In ‘Ode to a Nightingale’, Keats wrote these haunting lines: ‘Where youth grows pale, and spectre-thin, and dies’.^3^ In this article, building on my blog^4^ and a panel of TB book authors I moderated at the recent Union World Conference on Lung Health in Copenhagen,^5^ I spotlight a few excellent books (see Figure), broadly grouping these under four categories: historical; fiction; personal narratives; contemporary. This is by no means a comprehensive collection, but rather a subset that has inspired my work on TB.

**Figure.**
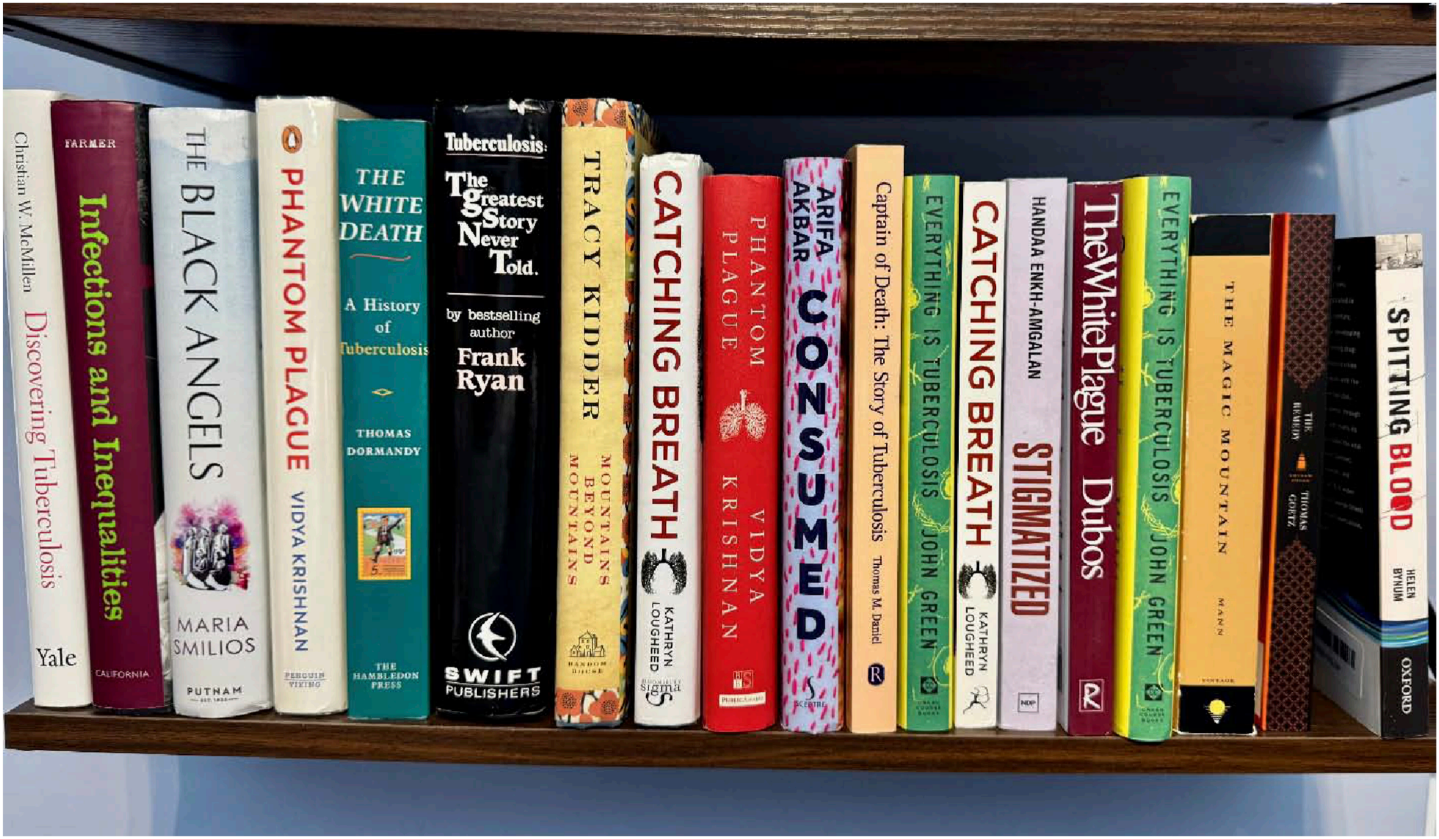
Over the centuries, several authors have written about TB and have helped us better understand the disease, its history, and its impact on people.

## HISTORY OF THE WHITE PLAGUE

Among historical books, ‘The White Plague: Tuberculosis, Man, and Society’ by René and Jean Dubos is a classic.^6^ TB treatment was not widely available when this book was written in 1952, but the authors were right on the spot on how social determinants have shaped the TB epidemic. They wrote, ‘TB presents problems that transcend the conventional medical approach…the impact of social and economic factors [must] be considered as much as the mechanisms by which tubercle bacilli cause damage to the human body’.^6^ Rene Dubos, a French-American soil microbiologist who trained under Selman Waksman at Rutgers University, lost his first wife, Marie Louise, to TB, which may have inspired him do work on this disease. Waksman, of course, won the 1952 Nobel Prize for his discovery of streptomycin.

‘Tuberculosis: The Greatest Story Never Told’ by Frank Ryan, a British physician and writer, was published in 1992.^7^ It is a hopeful story of how TB finally became a curable disease, thanks to the discovery of streptomycin, isoniazid, and other antibiotics. However, the book ended with the onset of the AIDS epidemic, and the growing threat of drug-resistant TB. Ryan called these an ‘alliance of terror’ and predicted that new ideas and new heroes would be needed to tackle the emerging crisis.

Since 1992, we know how the story evolved – AIDS became a global epidemic, with over 40 million people living with HIV in 2024, and made the TB epidemic much worse. And drug-resistant TB continues to be a global threat even today, with 390,000 people developing multidrug-resistant or rifampicin-resistant TB in 2024.^1^ Thomas Daniel’s ‘Captain of Death’ was published in 1997.^8^ Daniel was a TB clinician and is a retired professor of medicine at Case Western Reserve University. He wrote in his introduction, ‘Eight million people develop this disease every year’, and predicted correctly that TB ‘will almost certainly continue to increase’. Thomas Dormandy’s ‘The White Death’ offers a superb historical account and is full of old photographs and illustrations.^9^ Dormandy was a chemical pathologist in England. Written in 1999, his words still ring true: ‘Time and again in the past -–even before chemotherapy was supposed to have delivered the coup de grace – tuberculosis seemed conquered. Time and again it re-emerged with a grin’. ‘Spitting Blood’, published in 2012 by Helen Bynum, a British historian of science and medicine, is another book on the history of TB.^10^ ‘The story of tuberculosis is far from over’, she concluded as well.

In ‘The Remedy’ (2014), Thomas Goetz, a journalist and entrepreneur, chronicles the story of Robert Koch who not only identified the cause of TB but also worked hard to develop a cure for the disease.^11^ It also follows the life of Arthur Conan Doyle who, inspired by Koch’s approach, adapted his scientific methods into his stories featuring a detective named Sherlock Holmes. Until I read ‘The Remedy’, I had no idea that Koch, Doyle, and Holmes had any connection at all. A highly accessible book on the history of TB is ‘Tuberculosis: a short history’ edited by Alexander Medcalf, Henrice Altink, Monica Saavedra, and Sanjoy Bhattacharya, published by the University of York and Orient BlackSwan in 2013.^12^ The entire book, filled with historical photographs, can be downloaded for free.

It is fascinating that several of these historical books predicted that TB is a disease that is unlikely to disappear. They were spot on. In 2024, an estimated 10.7 million people fell ill with TB worldwide and 1.23 million people died of TB.^1^ I have no doubt that future historians and book authors will continue to write about TB.

## FICTION

While scores of fictional books and films include characters dying of TB,^4^ ‘The Magic Mountain’ is famous. This 1924 novel by Thomas Mann, a German novelist and Nobel laureate, first published with the original title of ‘Der Zauberberg’ in Germany, captures the experience of people in a TB sanatorium in Davos, Switzerland.^13^ Ironically, Davos now receives the rich and famous at the World Economic Forum every year.

There are characters in Dostoyevsky’s novels ‘Crime and Punishment’^14^ and ‘The Idiot’^15^ who suffered from TB. ‘The Jungle’^16^ by Upton Sinclair relates how Chicago’s meatpackers were exposed to TB as an occupational hazard, and ‘The Constant Gardener’^17^ by John Le Carré deals with a corporate scandal which involves unethical testing of an experimental TB drug in Africa. Of course, TB appears in several films, including the famous 2001 film ‘Moulin Rouge’, directed by Baz Luhrmann.

## PERSONAL STORIES

TB has deeply affected millions, and some have told their personal stories. In ‘Mountains Beyond Mountains’ (2003), American writer and Pulitzer prize winner, Tracy Kidder, traced the life of physician and anthropologist Paul Farmer, especially his inspiring work on fighting TB in Haiti and other countries.^18^ Farmer, who sadly passed away in 2022, himself had published ‘Infections and Inequalities: The Modern Plagues’ in 2001.^19^ The book uses TB and AIDS in Haiti to illustrate how these infections thrive on poverty and social determinants. My favourite line is at the very end of the book: ‘The poor, we’re told, will always be with us. If this is so, then infectious diseases will be, too’. Farmer’s legacy is alive today via the hundreds of dedicated people who work for Partners in Health, the NGO he founded along with Ophelia Dahl, Jim Yong Kim, Todd McCormack, and Thomas White. In ‘Consumed: A Sister’s Story’, Arifa Akbar, a journalist and theatre critic, wrote about the death of her older sister, Fauzia, who died of TB in London in 2016.^20^ The diagnosis had been missed by the medical team until after Fauzia had suffered a fatal intracerebral bleed. ‘Stigmatized’, published in 2021, is a memoir by Handaa Enkh-Amgalan, a Mongolian woman who was diagnosed with TB.^21^ The book chronicles her struggles with the disease, the stigma and social prejudice that often accompanies the disease, and her path to reclaiming her identify and agency. Enkh-Amgalan is now a leading TB advocate and speaker.

## CONTEMPORARY

‘Catching Breath: The Making and Unmaking of Tuberculosis’ (2017) is an exceedingly readable account of TB science, written by Katheryn Lougheed, a basic scientist who studied *Mycobacterium tuberculosis*.^22^ In ‘Phantom Plague’ (2022), Vidya Krishnan, a health and science journalist from India, traces the history of TB and connects that story to the ongoing impact it is having in India, the country with the highest number of people with TB.^23^ Although India has the resources to fight TB, too many people are still being left behind. Her book explains why. The ‘Black Angels’, published in 2023, is by Maria Smilios, an American author.^24^ Spanning the Great Depression and moving through World War II and beyond, this book is about the intrepid young women, the ‘Black Angels’, who, for 20 years, risked their lives working under dreadful conditions while caring for New York City’s poorest in a sanatorium called Sea View located in Staten Island. Despite their heroic work, these nurses were completely erased from history.

‘Everything is Tuberculosis’, published in 2025, is by John Green, a best-selling author of books including ‘The Fault in Our Stars’ and ‘Turtles All the Way Down’.^25^ The book is about the history of TB, and how it continues to devastate lives even today. Green dives deep into the structural inequities that allow TB to kill millions even today. ‘Tuberculosis is both a form and expression of injustice’, he writes.

At the 2025 Union World Conference on Lung Health in Copenhagen, authors of four contemporary books, John Green, Vidya Krishnan, Handaa Enkh-Amgalan, and Maria Smilios were celebrated for their inspiring work.^5^ In a highly rated panel discussion, they spoke about how their books demonstrate that TB thrives on racism, prejudice, poverty, and injustice.^5^ As they emphasised, if we wanted to end TB, we can. We are choosing not to.

I hope some day in the future, in my lifetime, a new book will tell the epic tale of how the white plague was finally defeated. That is the book I’ve been wanting to read forever.
